# Impact of binding site waters on inhibitor design: contemplating a novel inverse binding mode of indirubin derivatives in DYRK kinases

**DOI:** 10.1186/1758-2946-6-S1-P6

**Published:** 2014-03-11

**Authors:** Daniel Cappel, Vassilios Myrianthopoulos, Emmanuel Mikros, Woody Sherman

**Affiliations:** 1 Schrödinger GmbH, Dynamostraße 13, 68165 Mannheim, Germany; 2 University of Athens, Panepistimiopolis Zografou, 15771 Athens, Greece; 3 Schrödinger Inc., 120 West 45 th Street, 17 th Floor, New York NY 10036, USA

## 

DYRK kinases are involved in alternative pre-mRNA splicing as well as in neuropathological states such as Alzheimer's disease and Down syndrome. In this study, we present the design, synthesis, and biological evaluation of indirubins as DYRK inhibitors with enhanced selectivity. Modifications of the bis-indole included polar or acidic functionalities at positions 5′ and 6′ and a bromine or a trifluoromethyl group at position 7, affording analogues that possess high activity and pronounced specificity. Compound 6i carrying a 5′- carboxylate moiety demonstrated the best inhibitory profile. A novel inverse binding mode, which forms the basis for the improved selectivity, was suggested by molecular modeling and confirmed by determining the crystal structure of DYRK2 in complex with 6i. Structure–activity relationships were further established, including a thermodynamic analysis of binding site water molecules, offering a structural explanation for the selective DYRK inhibition [1].
